# Caregiving and Its Resulting Effects—The Care Study to Evaluate the Effects of Caregiving on Caregivers of Patients with Advanced Cancer in Singapore

**DOI:** 10.3390/cancers8110105

**Published:** 2016-11-15

**Authors:** Cheryl Kai Ting Chua, Jun Tian Wu, Yin Yee Wong, Limin Qu, Yung Ying Tan, Patricia Soek Hui Neo, Grace Suyin Pang

**Affiliations:** 1 National University Health System, Singapore 119228, Singapore; 2 Health Services Research Unit, Division of Research, Singapore General Hospital, Singapore 169608, Singapore; wu.jun.tian@sgh.com.sg; 3 Clinical Quality Department, Assisi Hospice, Singapore 574623, Singapore; wong.yin.yee@assisihospice.org.sg; 4 Department of Palliative Care, National Cancer Centre Singapore, Singapore 169610, Singapore; qu.l.m@nccs.com.sg (L.Q.); tan.yung.ying@nccs.com.sg (Y.Y.T.); patricia.neo.s.h@singhealth.com.sg (P.S.H.N.); 5 Care Integration Division, Agency for Integrated Care, Singapore 069110, Singapore

**Keywords:** caregiving, advanced cancer, burden, quality of life, depression, work impairment

## Abstract

Informal caregivers (IC) are key to enabling home deaths, where preferred, at the end-of-life. Significant morbidity from advanced cancer can make caregiving burdensome. However, knowledge about the nature of the caregiving burden for caregivers in Singapore is limited. Hence, the key objective in this study was to examine the impact of the caregiving burden on quality of life (QOL), mental health and work capacity among local ICs. Eligible English-speaking ICs of hospitalized advanced cancer patients were recruited through non-random sampling. The Zarit Burden Interview (ZBI), Caregiver Quality of Life Index—Cancer (CQOLC), Center for Epidemiologic Studies Depression Scale—Revised (CESD-R), and Work Productivity and Activity Impairment Questionnaire (WPAI) were interviewer-administered to eligible ICs. Altogether, 16 ICs were surveyed. The mean age of ICs was 43.8 years. Most were children of patients (43.8%), and eight ICs had high burden (ZBI > 17). Those with ZBI > 17 had lower QOL, higher depression scores as well as greater work and activity impairment. In conclusion, high caregiver burden has adverse effects on QOL, mental health and work productivity. Non-physical elements of caregiving (particularly financial and decision-making) and increased number of care roles undertaken by a single IC contribute to high burden. Future interventions for caregiving burden in Singapore should also address the financial and decision-making aspects of caregiving. Outsourcing selected aspects of the caregiving role to community services may reduce the number of caregiving aspects undertaken by a single IC and caregiver burden.

## 1. Introduction

Caregiver burden is the description of a negative reaction to the impact of care on an informal caregiver’s (IC’s) social, occupational and personal roles. These reactions encompass the ICs’ response to physical, psychological, social and financial challenges and can be prolonged in chronic or mental illness [1]. Caring for patients with advanced cancer can thus be burdensome for ICs, as advanced cancer is associated with significant morbidity. Outside Singapore, high caregiver burden has been previously reported to be associated with negative effects on physical, mental health and general well-being [2,3,4,5]. However, most studies in cancer caregiving have focused either on burden alone or on selected caregiving outcomes. To the best of our knowledge, no study has examined burden and multiple caregiving outcomes in the same setting for advanced cancer in Singapore.

Singaporean society was built upon Confucianism principles, with an emphasis on filial piety [6,7]. Hence, the family is the first line of support for patients with family members expected to take on the role of informal caregiving [8]. Family ICs can be involved in multiple aspects of informal caregiving and often act as proxy decision makers for patients in medical decision-making [9]. However, urbanization has produced increasingly smaller families, thus reducing the number of available family caregivers and limiting the extent to which the burden can be shared between family members [10].

Singapore’s healthcare financing model is based on co-payment between the individual and state. Subsidies are given to patients post-means testing based on household headcount and income criteria. For patients receiving low or no subsidies, the out-of-pocket payment can be a heavy financial burden. Notably, in 2014, 94.1% of private expenditure on health was out-of-pocket for patients in Singapore, in contrast to other nations such as the United States (21.4%), United Kingdom (57.7%), European Union (62.9%) and Australia (57.1%) [11,12]. The financial burden is further amplified when the caregiving role causes disruption to one’s employment. Results from a survey in Singapore of general family caregivers showed that half of caregivers were in the economically active group aged below 50 years, one in five caregivers gave up their jobs to care for aged relatives and one in four reported a worsening of financial position after taking on the caregiving role [13].

Hence, our study aimed to describe the quality of life (QOL), economic and mental health aspects of caregiver burden in ICs of patients with advanced cancer in Singapore, as well as to identify potential contributory factors to the burden.

## 2. Materials and Methods

Institutional Review Board approval was obtained before commencing this study. ICs in this study were defined as caregivers providing unpaid care to the patient. Non-random sampling was used to identify suitable caregivers.

### 2.1. Eligibility Criteria

The inclusion criteria were:

- ICs of advanced cancer patients admitted to Singapore General Hospital (SGH) within 3 weeks of the interview date and receiving care from the National Cancer Centre Singapore (NCCS) palliative care service.
- Significant involvement in at least one of four care roles: physical care, financial support, emotional support and decision making, with significant involvement defined as undertaking at least 50% of the care role as determined by the IC.
- Singaporeans or Singapore permanent residents.
- Aged 21 years old and above.
- Able to communicate in English.
- Intact cognition.

**Figure 1:**
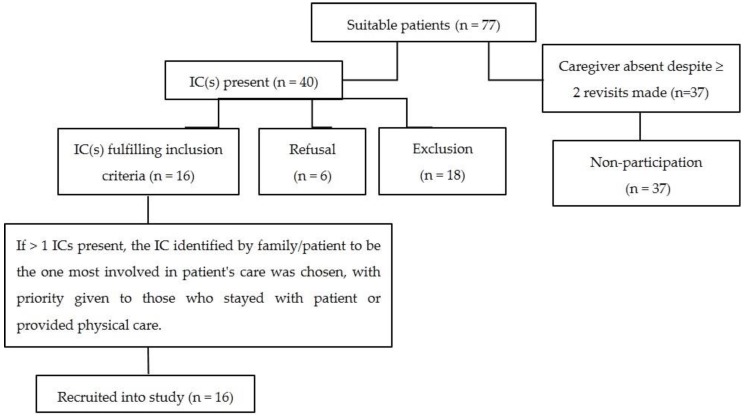
Methodology.

ICs of patients deemed unsuitable based on the clinical judgment of the palliative care team in charge were excluded. ICs could choose to be surveyed in the presence of or away from the patient. Demographical information, specific information about the caregiving role and clinical data related to the ICs, as well as patient-related information from the patient’s medical notes were collected as part of the study. Figure 1 summarizes the recruitment process of this study.

### 2.2. Surveys Administered

The following surveys were administered once to ICs, with the help of the interviewer:

- Palliative care Outcomes Scale (POS)—Carer questionnaire. The POS is a 10-item scale with scores varying proportionately to the level of patients’ physical symptoms, psychological, emotional, spiritual, information and support needs. The outcome measure is developed for patients with advanced cancer and has been validated in various palliative care settings (both inpatient and outpatient). Its construct has been validated against other appropriate palliative care measures such as the European Organisation for Research on Cancer Treatment and the Support Team Assessment Schedule [14].
- Zarit Burden Interview (ZBI)—short-form 12-item version. In this 12-item short version of the original 22-item ZBI, higher scores represent higher burden. The ZBI is a widely used tool to describe caregiver burden and has been well validated in various caregiving samples. The scores from the 12-item version have previously been assessed to have good correlation with the original version in caregivers of advanced cancer patients. A score of 17 or more has been recommended as an indicator for high caregiver burden [15,16].
- Caregiver Quality of Life Index—Cancer (CQOLC). Higher scores correspond to better QOL in this 35-item questionnaire. Psychometric properties have previously been reported with test-retest reliability of 0.95, good internal consistency and demonstration of face, content, concurrent, convergent as well as discriminant validity among family caregivers of cancer patients [16,17,18].
- Center for Epidemiologic Studies Depression Scale—Revised (CESD-R). A score of 16 or above has been considered clinically significant in this 20-item depression scale reflecting the Fourth Edition of the Diagnostic and Statistical Manual of Mental Disorders (DSM-IV) criteria for depression [19].
- Work Productivity and Activity Impairment Questionnaire (WPAI). The proportion of work and activity impairment is assessed through 6 questions pertaining to work and non-work activities. The WPAI has previously reported to have high convergent validity with the Caregiver Strain Index and the Centre for Epidemiologic Studies Depression Scale in ICs [20].

Information related to caregiver demographics and caregiving tasks was also collected from the surveyed ICs. SPSS V2 was used for data analysis. Data was described using measures of frequency as well as central tendency such as the mean and median.

## 3. Results

### 3.1. Participants

A total of 16 ICs were surveyed (Figure 1). Most ICs (62.5%) were interviewed by patients’ bedside whilst the remainder chose to be interviewed away from the patient. The demographic profiles of surveyed ICs and patients are listed in Table 1. ICs were about 20 years younger than the patients (43.8 years versus 63 years respectively) with more children than spouses undertaking the IC role (43.8% versus 25% respectively). Most ICs (68.8%) reported no chronic medical conditions.

Characteristics of the caregiving context (Table 2) showed that 81.3% of all ICs provided care daily. One-third had significant involvement in all four caregiving roles of physical care, emotional care, decision-making and financial support. Only a minority received support in the form of foreign domestic worker help (31.3%), financial support (25%) or homecare services (18.8%). Only one IC had prior caregiver training.

Table 3 summarizes the profile of patients cared for by study ICs. The commonest cancer diagnoses in patients cared for were of the gastrointestinal type. Patients who had been diagnosed with cancer were an equal mix between those with long (≥2 years since cancer diagnosis) and short cancer durations.

### 3.2. Burden of Caregiving

Table 4 shows burden and outcomes of burden. Half of ICs had a ZBI score above the recommended threshold of 17 for diagnosis of high caregiver burden [15]; mean total score was 17.81 (9.98). Relative to the maximum domain score, the ZBI domain score was highest in the area of burden in relationship (3.56 (1.93) out of 8), followed by personal strain (10.63 (5.27) out of 24). The mean CQOLC score of ICs was 81.19 (25.24). The CQOLC subscale score was lowest (implying poorer QOL) in the subscale of burden (16.25 (10.1) out of 40). The mean depression score on the CESD-R was 18.69; this was higher than the recommended threshold of 16 to define depression. Mean work impairment scores of 57.85% (35.24) on WPAI implied that ICs experienced more than 50% impairment of their work.

ICs with ZBI ≥ 17 showed lower scores for QOL (CQOLC 62.25 vs. 100.13), depression (CESD-R 18.38 vs. 10.13) and greater impairment in work capacity (79.0% vs. 45.0%), compared to those with ZBI < 17 respectively (Table 4).

Higher ZBI scores were observed among ICs who balanced multiple responsibilities, experienced significant physical and non-physical caregiving demands, cared for patients with debilitating symptoms or had poor social support (Table 5).

## 4. Discussion

Much attention has been placed on the fact of home being the preferred place of patients particularly near the end-of-life (EOL). The sudden and major news of cancer often results in ICs entering their caregiving role barely prepared for the challenges they may encounter [21]. Their needs are also often overlooked and easily dismissed as more focus is usually placed on the patients. Our results reveal considerable burden among Singaporean ICs, with those experiencing high burden concurrently facing reduced QOL, higher risks of depression and increased work and activity impairment.

The burden in caregiving has negative effects on ICs’ physical, emotional, social and financial well-being and overall QOL. Our study’s mean CQOLC score was similar to the score of 83.5 previously reported in a study of 258 Singaporean cancer caregivers [22]. The same study revealed that ICs in Singapore and Asia (Iran, South Korea, Turkey, Taiwan) had lower QOL as compared to their Western counterparts (United States, United Kingdom, Canada) and that QOL was worst among ICs who cared for advanced-stage cancer [22]. Our study further shows that QOL is considerably lower among ICs experiencing high burden.

In addition to the more commonly known physical demands of caregiving, providing care to a terminally ill cancer patient is also mentally burdensome. Depression affects 5.8% of Singapore’s population. Caring for a sick relative has been shown to confer increased risk of mental disorders in the Singapore Mental Health Study [23]. Our findings are consistent with studies of caregivers outside Singapore. Asian ICs have been found to be at high risk for depression and care burden has been observed to be a predictor of depression among cancer ICs [7,24]. Physicians thus need to be aware of the emotional and psychological problems that ICs may battle with, be able to identify those at risk of psychological illnesses and implement preventive strategies and timely interventions. This may be challenging in view of the existing stigma associated with mental disorders, as well as the tendency of ICs to neglect their own health or hide issues surrounding their health [25,26,27].

Work is integral in the lives of most middle-aged people belonging to the “sandwich” generation. In the context of caregiving, work provides not just economic security but may also offer respite and social support to ICs. Work impairment contributes to the financial burden of caregiving and has both personal and social implications for the IC. With more employees projected to be involved in caregiving, loss of productivity and efficacy may invariably result in economic consequences at the level of companies and the nation. Work impairment in our study population was substantial and higher than that in European and American ICs (46.3% and 22.88% respectively) [4,28]. This could be due to the differences in profiles of ICs (younger and more likely to be a child of the patient in Singapore), as well as lower availability of feasible flexible work arrangements [29,30]. In 2014, 1 in 10 Singapore residents aged 25–54 years old cited caregiving as the main reason for not working or seeking employment [30]. This was despite 47% of firms providing at least one formal flexible work arrangement. Part-time work was most commonly offered (36% of companies), whereas other arrangements such as flexible work hours, staggered hours, tele-working were less often offered (12%, 11% and 5.8% respectively) [31].

Previously cited determinants of high burden include ICs’ poor health, lack of family or social support, financial difficulties, and caring for patients requiring high intensity of assistance or with greater symptom distress [5,32]. In addition to the physical demands of caregiving, our study also reveals that non-physical elements are equally critical in generating burden, particularly in areas of financial care, surrogate decision-making and in juggling multiple responsibilities.

Healthcare financing in Singapore is a shared responsibility between the state and the patient or his family. Caregiving studies in Singapore have revealed that ICs face worsening financial situations and those facing financial problems have high risks of negative outcomes of stress and depression [13,33]. In an informal survey conducted on Singaporean ICs caring for aged care recipients, ICs reported that they were at least 50% certain that care recipients’ medical expenses would deplete their Medisave and non-Medisave savings [33]. Our study shows that advanced cancer ICs who provided financial care face higher burden. This burden may be related to worries they may have about their financial adequacy to support their relatives’ medical care. Further studies into the financial worries and difficulties faced by ICs who provide financial care to advanced cancer patients will be useful.

ICs are often tasked to assist in making decisions about patients’ medical care. Such decisions may be about the extent of aggressive interventions in the event of medical deterioration, do-not-resuscitate (DNR) orders, and preferred place of terminal care and demise. For a trained physician, such decisions are challenging and thus even more so for the non-medically trained IC. Existing literature on the stress implicated in surrogate decision-making is limited, with most studies focusing on the concordance between surrogates’ decisions and patients’ preferences. Nevertheless, reduction in burden of decision-making has been cited as a perceived benefit of advanced care planning (ACP) [34]; this is likely related to improved familiarity of surrogates with patients’ EOL preferences. Further studies into the stress in surrogate decision-making among cancer patients will be useful.

ICs surveyed in our study were mainly children caring for their parents, differing from the usual spousal relationship we observe in most caregiving studies especially in Western nations [35], and may represent the anticipated shift towards adult children taking on caregiving roles with a corresponding reduction in proportion of spousal caregivers [36]. Characteristics of ICs facing high burden suggest the vulnerability of the “sandwich” generation, caught between the competing demands of their careers, caring for families and aged parents. With an average family size of 3.39 [37], the small Singaporean family unit forces Singaporean ICs to take up multiple caregiving roles, putting them at increased vulnerability to the stress arising from multiple responsibilities.

### Strengths and Limitations

This study was designed to be exploratory in nature, intended to bring into perspective a broad coverage of caregiving and its resulting effects, and identify problems of caregiving pertinent to the local Singapore population.

We recognize that our study was limited to the small sample size obtained through non-random sampling that selected for ICs who were English speaking and were available to be surveyed. The small sample size limited to inpatients potentially excluded other caregiving situations faced by ICs. We also recognize the potential for interviewer bias in this study.

These limitations were considered but also weighed against the breadth of data we sought to obtain in this study. Scales used in our study were all available and studied in their English form but versions in Mandarin—a common language among Singaporean caregivers—had not all been validated. We were aware that some caregivers might be afraid to leave their loved ones unattended to be interviewed away from the patient and hence did not enforce a consistent interview setting away from the patient in a bid to minimize bias.

## 5. Conclusions

In conclusion, our study shows that family ICs face considerable burden when caring for patients with advanced cancer, with resultant adverse effects on their work, quality of life and mental health. Our pilot study identifies less commonly discussed contributors of burden that are potentially modifiable, specifically, the non-physical aspects of caregiving such as psycho-emotional stress, decision-making, financial factors or the juggling of multiple caregiving responsibilities. Future studies to validate our findings may be useful and should be designed with a view to develop appropriate strategies to support and empower ICs to provide care.

## Figures and Tables

**Table 1:** Sample characteristics.

Socio-Demographics		Caregivers	Patients
Mean age in years (S.D.)		43.8 (14.80)	63.0 (17.51)
Gender	Male, n (%)	7 (43.8%)	8 (50%)
	Female	9 (56.3%)	8 (50%)
Ethnicity	Chinese	11 (68.8%)	
	Malay	1 (6.3%)	
	Indian	3 (18.8%)	
	Others	1 (6.3%)	
Marital status	Single	6 (37.5%)	
	Married	10 (62.5%)	
Number of children <16 years	0	13 (81.3%)	
	1	1 (6.3%)	
	2	2 (12.5%)	
Employment status	Employed	8 (50%)	
	Professional, managerial	5 (31.3%)	
	Clerical, service, sales	2 (12.5%)	
	Manual skilled or unskilled	1 (6.3%)	
	Unemployed	8 (50%)	

S.D., standard deviation.

**Table 2:** Context of caregiving.

Caregiving Variables		n (%)
Caregiver-patient relationship	Spouse	4 (25.0)
	Child	7 (43.8)
	Sibling	2 (12.5)
	Extended family	3 (18.8)
Duration of care	<6 months	5 (31.3)
	6 months–1 year	1 (6.3)
	1–2 years	2 (12.5)
	≥2 years	8 (50)
Amount of involvement in daily care	No	3 (18.8)
	Yes	13 (81.3)
	<4 h	0
	4–8 h	1 (7.7)
	8–12 h	2 (15.4)
	≥12 h	10 (76.9)
Number of caregiving roles	1	2 (12.5)
	2	5 (31.3)
	3	4 (25.0)
	4	5 (31.3)
Physical caregiving	No	2 (12.5)
	Yes ^1^	8 (50.0)
	Partially ^2^	1 (6.3)
	N/A ^3^	5 (31.3)
Hours spent on physical caregiving	<4 h	1 (11.1)
	4–8 h	3 (33.3)
	8–12 h	0
	≥12 h	5 (55.6)
Financial caregiving	No	3 (18.8)
	Yes ^1^	5 (31.3)
	Partially ^2^	5 (31.3)
	N/A ^3^	3 (18.8)
Emotional caregiving	No	1 (6.3)
	Yes ^1^	12 (75.0)
	Partially ^2^	3 (18.8)
Decision-making	No	1 (6.3)
	Yes ^1^	6 (37.5)
	Partially ^2^	4 (25.0)
	N/A ^3^	5 (31.3)
Employment of foreign domestic worker	No	11 (68.8)
	Yes	5 (31.3)
	<6 months	1 (20.0)
	6 months–1 year	1 (20.0)
	1–2 years	1 (20.0)
	≥2 years	2 (40.0)
Home care service	No	13 (81.3)
	Yes	3 (18.8)
Financial assistance	No	12 (75.0)
	Yes	4 (25.0)
Attended caregiver training	No	15 (93.8)
	Yes	1 (6.3)
Previous experience in caring for someone sick	No	11 (68.8)
	Yes	5 (31.3)

^1^ “Yes” referring to at least 50% involvement in the caregiving role; ^2^ “Partially” referring to less than 50% involvement in the caregiving role; ^3^ “N/A” or “not applicable” implies that the patient did not require care in this aspect.

**Table 3:** Patients’ health status.

Variables		n (%)
Cancer type	Breast	3 (18.8)
	Digestive or gastrointestinal (gastric, colorectal, liver)	6 (37.5)
	Head and neck (thyroid)	1 (6.3)
	Genitourinary (bladder, prostate, renal)	3 (18.8)
	Gynecological (ovarian)	1 (6.3)
	Hematological (blood, lymphoma)	1 (6.3)
	Respiratory (lung)	1 (6.3)
Time since diagnosis	<6 months	5 (31.3)
	6 months–1 year	1 (6.3)
	1–2 years	2 (12.5)
	≥2 years	8 (50)
Distant metastasis	Yes	15 (93.8)
	No	1 (6.3)
Time since admission	<1 week	12 (75.0)
	1–2 weeks	3 (18.8)
	2–3 weeks	1 (6.3)
Caregiver rated KPS ^1^	80–100	3 (18.8)
	50–70	8 (50.0)
	0–40	5 (31.3)
POS—Carer ^2^ total score	≤10	1 (6.3)
	10–20	7 (43.8)
	>20	8 (50.0)

^1^ KPS: Karnofsky Performance Status; ^2^ POS: Palliative care Outcome Scale—Carer questionnaire.

**Table 4:** Outcomes of caregiving.

Caregiving Outcomes		Possible Range	Mean (SD)		
			All, n = 16	ZBI < 17, n = 8	ZBI ≥ 17, n = 8
Caregiver burden	ZBI ^1^ total score	0–48	17.81 (9.98)		
ZBI ^1^ domains	Burden in relationship	0–8	3.56 (1.93)		
	Emotional well-being	0–20	7.19 (4.79)		
	Social and family life	0–12	4.19 (2.64)		
	Loss of control over one’s life	0–8	2.88 (2.25)		
	Personal strain	0–24	10.63 (5.27)		
	Role strain	0–20	6.5 (4.07)		
Caregiver QOL	CQOLC ^2^ total score	0–140	81.19 (25.24)	100.13 (14.42)	62.5 (18.38)
CQOLC ^2^ subscales	Burden	0–40	16.25 (10.10)		
	Disruptiveness	0–28	18.44 (6.48)		
	Positive adaptation	0–28	20.38 (4.76)		
	Financial concerns	0–12	8.63 (3.32)		
Caregiver depression	CESD-R ^3^ total score	0–60	18.69 (12.65)	10.13 (7.02)	27.25 (11.24)
WPAI ^4^	Proportion of absenteeism, %	0–100	41.04 (37.21)		
	Proportion of presenteeism-related impairment, %	0–100	41.25 (38.71)		
	Overall work impairment, %	0–100	57.85 (35.24)	45.00 (38.40)	79.00 (18.10)
	Activity impairment, %	0–100	47.5 (27.45)	32.50 (27.12)	62.50 (19.01)

^1^ ZBI: Zarit Burden Interview; ^2^ CQOLC: Caregiver Quality of Life Index—Cancer; ^3^ CESD-R: Center for Epidemiologic Studies Depression Scale—Revised; ^4^ WPAI: Work productivity and activity impairment Questionnaire.

**Table 5:** Caregiver and patient characteristics observed in caregivers with high caregiver burden.

**Characteristics of Caregivers**			
Balancing multiple responsibilities	Physical caregiving demands	Non-physical aspects of caregiving	Social support
Aged 31–50 years Had a child/children <16 years Involved in all 4 caregiving roles	Physical caregiving ≥12 h spent on physical caregiving	Spousal relationship with patient Caring for ≥2 years Providing financial care Making decisions pertaining to the care of the patient	Lack of:
			Foreign domestic helper
			Home care service
			Financial assistance

**Patient Characteristics**			
Physical demands	Symptoms		
Male patients	High caregiver-rated POS score of >20 Distant metastasis to bones and/or lungs		

## Abbreviations

The following abbreviations are used in this manuscript:

IC: Informal caregiverQOL: Quality of life
SGH: Singapore General Hospital
NCCS: National Cancer Centre Singapore
POS: Palliative care Outcome Scale
ZBI: Zarit Burden Interview
CQOLC: Caregiver Quality of Life Index—Cancer
CESD-R: Center for Epidemiologic Studies Depression Scale—Revised
DSM-IV: Fourth Edition of the Diagnostic and Statistical Manual of Mental Disorders
WPAI: Work Productivity and Activity Impairment Questionnaire
SD: Standard deviation
KPS: Karnofsky Performance Status
EOL: End-of-life
DNR: Do-not-resuscitate
ACP: Advanced care planning

## References

[1] Chan S.W. (2010). Family caregiving in dementia: The Asian perspective of a global problem. Dement. Geriatr. Cogn. Disord..

[2] Hebert R.S., Schulz R. (2006). Caregiving at the end of life. J. Palliat. Med..

[3] Turkoglu N., Kilic D. (2012). Effects of care burdens of caregivers of cancer patients on their quality of life. Asian Pac. J. Cancer Prev..

[4] Goren A., Gilloteau I., Lees M., DaCosta Dibonaventura M. (2014). Quantifying the burden of informal caregiving for patients with cancer in Europe. Support Care Cancer.

[5] Lee K.C., Chang W.C., Chou W.C., Su P.J., Hsieh C.H., Chen J.S., Tang S.T. (2013). Longitudinal changes and predictors of caregiving burden while providing end-of-life care for terminally ill cancer patients. J. Palliat. Med..

[6] Ng H.Y., Griva K., Lim H.A., Tan J.Y., Mahendran R. (2016). The burden of filial piety: A qualitative study on caregiving motivations amongst family caregivers of patients with cancer in Singapore. Psychol. Health.

[7] Tang S.T., Li C.Y., Liao Y.C. (2007). Factors associated with depressive distress among Taiwanese family caregivers of cancer patients at the end of life. Palliat. Med..

[8] Leow M.Q., Chan M.F., Chan S.W. (2014). Predictors of change in quality of life of family caregivers of patients near the end of life with advanced cancer. Cancer Nurs..

[9] Searight H.R., Gafford J. (2005). Cultural diversity at the end of life: Issues and guidelines for family physicians. Am. Fam. Physician.

[10] Households and Housing. http://www.singstat.gov.sg/statistics/latest-data#22.

[11] Out-of-Pocket Health Expenditure (% of Private Expenditure on Health). http://data.worldbank.org/indicator/SH.XPD.OOPC.ZS.

[12] WHO Guide to Identifying the Economic Consequences of Disease and Injury. http://www.who.int/choice/publications/d_economic_impact_guide.pdf.

[13] Ng G.T. Study Report of Singapore Family Caregiving Survey. http://www.fas.nus.edu.sg/rg/doc/family/family_wp.pdf.

[14] Hearn J., Higginson I.J. (1999). Development and validation of a core outcome measure for palliative care: The palliative care outcome scale. Palliative Care Core Audit Project Advisory Group. Qual. Health Care.

[15] Higginson I.J., Gao W., Jackson D., Murray J., Harding R. (2010). Short-form Zarit Caregiver Burden Interviews were valid in advanced conditions. J. Clin. Epidemiol..

[16] Whalen K.J., Buchholz S.W. (2010). The reliability, validity and feasibility of tools used to screen for caregiver burden: A systematic review. J. Adv. Nurs..

[17] Weitzner M.A., Jacobsen P.B., Wagner H.J., Friedland J., Cox C. (1999). The Caregiver Quality of Life Index-Cancer (CQOLC) scale: Development and validation of an instrument to measure quality of life of the family caregiver of patients with cancer. Qual. Life Res..

[18] Weitzner M.A., McMillan S.C. (1999). The Caregiver Quality of Life Index-Cancer (CQOLC) Scale: Revalidation in a home hospice setting. J. Palliat. Med..

[19] Van Dam N.T., Earleywine M. (2011). Validation of the Center for Epidemiologic Studies Depression Scale—Revised (CESD-R): Pragmatic depression assessment in the general population. Psychiatry Res..

[20] Giovannetti E.R., Wolff J.L., Frick K.D., Boult C. (2009). Construct validity of the Work Productivity and Activity Impairment questionnaire across informal caregivers of chronically ill older patients. Value Health.

[21] Glajchen M. (2004). The emerging role and needs of family caregivers in cancer care. J. Support Oncol..

[22] Lim H.A., Tan J.Y.S., Chua J., Yoong R.K.L., Lim S.E., Kua E.H., Mahendran R. (2016). Quality of life of family caregivers of cancer patients in Singapore and globally. Singapore Med. J..

[23] Chong S.A., Abdin E., Vaingankar J.A., Heng D., Sherbourne C., Yap M., Lim Y.W., Wong H.B., Ghosh-Dastidar B., Kwok K.W. (2012). A population-based survey of mental disorders in Singapore. Ann. Acad. Med. Singapore.

[24] Rhee Y.S., Yun Y.H., Park S., Shin D.O., Lee K.M., Yoo H.J., Kim J.H., Kim S.O., Lee R., Lee Y.O. (2008). Depression in family caregivers of cancer patients: The feeling of burden as a predictor of depression. J. Clin. Oncol..

[25] Lai Y.M., Hong C.P.H., Chee C.Y.I. (2000). Stigma of Mental Illness. Singapore Med. J..

[26] National Mental Health Literacy Study. https://www.imh.com.sg/uploadedFiles/Newsroom/News_Releases/6Oct15_Mind%20Matters%20Media%20Release.

[27] Chang H.Y., Chiou C.J., Chen N.S. (2010). Impact of mental health and caregiver burden on family caregivers’ physical health. Arch. Gerontol. Geriatr..

[28] Mazanec S.R., Daly B.J., Douglas S.L., Lipson A.R. (2011). Work productivity and health of informal caregivers of persons with advanced cancer. Res. Nurs. Health.

[29] Labour Force in Singapore, 2014. http://stats.mom.gov.sg/Pages/Labour-Force-In-Singapore-2014.aspx.

[30] McGuire J.F., Kenney K., Brashler P. Flexible Work Arrangements: The Fact Sheet. http://scholarship.law.georgetown.edu/cgi/viewcontent.cgi?article=1012&context=legal.

[31] Xue J.Y. More companies offering flexible work arrangements: MOM survey. http://www.todayonline.com/singapore/more-companies-offering-flexible-work-arrangements-mom-survey.

[32] Dumont S., Turgeon J., Allard P., Gagnon P., Charbonneau C., Vézina L. (2006). Caring for a loved one with advanced cancer: Determinants of psychological distress in family caregivers. J. Palliat. Med..

[33] Chan A., Ostbye T., Malhotra R., Hu A.J. (2013). The Survey on Informal Caregiving.

[34] Ng R., Chan S., Ng T., Chiam A., Lim S. (2013). An exploratory study of the knowledge, attitudes and perceptions of advance care planning in family caregivers of patients with advanced illness in Singapore. BMJ Support. Palliat. Care.

[35] Romito F., Goldzweig G., Cormio C., Hagedoorn M., Anderson B.L. (2013). Informal caregiving for cancer patients. Cancer.

[36] Kim Y., Given B.A. (2008). Quality of life of family caregivers of cancer survivors: Across the trajectory of the illness. Cancer.

[37] Census of population 2010 Statistical Release 1: Demographic Characteristics, Education, Language and Religion. https://www.singstat.gov.sg/docs/default-source/default-document-library/publications/publications_and_papers/cop2010/census_2010_release1/cop2010sr1.pdf.

